# Secretion of interleukin-6 by bone marrow mesenchymal stem cells promotes metastasis in hepatocellular carcinoma

**DOI:** 10.1042/BSR20170181

**Published:** 2017-07-21

**Authors:** Fei Mi, Liansheng Gong

**Affiliations:** 1 Hepatobiliary and Enteric Surgery Research Center, Xiangya Hospital, Central South University, Changsha 410008, P.R. China; 2 Department of Finance and Trade, City College of Dongguan University of Technology, Dongguan 523419, P.R. China; 3 Department of Pancreatic Surgery, Xiangya Hospital, Central South University, Changsha 410008, P.R. China

**Keywords:** hepatocellular carcinoma, interleukin-6, mesenchymal stem cell, metastasis, STAT3

## Abstract

Mesenchymal stem cells (MSCs) interact with tumor cells and regulate tumorigenesis and metastasis. As one of the important components of the tumor microenvironment, MSC-secreted cytokines play a critical role in cancer development. However, whether and how bone marrow MSCs (BMSCs) and their secreted cytokines participate in hepatocellular carcinoma (HCC) progression, still remains largely unknown. In the present study, we first measured the concentration of interleukin-6 (IL-6) in BMSC conditioned medium (BMSC-CM). Next, we assessed the changes of invasion ability in response to treatment of BMSC-CM or recombinant IL-6 in two human HCC cell lines Bel-7404 and HepG2. Then we analyzed the level of key components of the IL-6 signal pathway, including IL-6 receptor and signal transducer (i.e. IL-6R and gp130), a transcription factor STAT3 (signal transducer and activator of transcription 3), as well as its target genes BCL2, CCND1, MCL1 and MMP2, in BMSC-CM or recombinant IL-6 treated Bel-7404 and HepG2 cells. Results showed that a considerable amount of IL-6 was secreted by BMSCs, and BMSC-CM markedly elevated Bel-7404 cell invasion rate and stimulated the signal transduction of IL-6/STAT3 pathway. Neutralizing the secreted IL-6 bioactivity by the anti-IL-6 antibody diminished the invasion-promoting effect and down-regulated IL-6/STAT3 pathway of BMSC-CM treated Bel-7404 cells. In conclusion, we found that BMSCs may activate the IL-6/STAT3 signaling pathway and promote cell invasion in Bel-7404 cells, suggesting that this protumor effect should be seriously considered before clinical application of MSC-mediated cancer therapy.

## Introduction

Mesenchymal stem cells (MSCs) can interact with tumor cells, directly and/or indirectly and affect the development of multiple types of cancer [1,2]. They are recruited to tumorigenic sites and secrete factors such as interleukin-6 (IL-6), interleukin-10 (IL-10), vascular endothelial growth factor (VEGF) etc. These secretions have been proved to have antitumor and/or protumor effects [3]. Therefore, the MSC-derived conditioned medium (CM) (MSC-CM) has opposing effects on tumor development owing to different sources of the MSCs, compositions of the secreted factors or the biological context. Emerging evidence have emerged to demonstrate the functions and regulatory mechanisms of MSC-CM in various cancer types [4,5]. However, it has not been fully understood how human bone marrow MSC (BMSC)-CM affects hepatocellular carcinoma (HCC) cell properties, e.g. migration, invasion, and proliferation.

As a collection of secreted cytokines and growth factors, MSC-CM plays a critical role in regulating tumor initiation and progression by affecting the invasion, migration, or apoptosis resistance of tumor cells, positively or negatively [3]. Amongst these secreted factors, IL-6, a pro-inflammatory cytokine, has been proven to contribute to the metastatic cancers, including HCC [6], gastric cancer [7], breast cancer [8], and lung cancer [9]. Previous studies showed that BMSC produces a significant amount of IL-6, as well as IL-8, prostaglandin E2 (PGE2), and VEGF [10]. Considering the complexity of the multifunctional composition of BMSC-CM, it should be further addressed whether BMSC-CM regulates HCC through an IL-6-dependent pathway.

IL-6 stimulates several signaling pathways by binding to its specific receptor, which contains an α-chain subunit IL-6 receptor (IL-6R) and a gp130 (IL-6 signal transducer) subunit. The binding of IL-6 to the receptor can stimulate downstream signals, including STAT1 (signal transducer and activator of transcription 1), SOCS3 (suppressor of cytokine signaling 3), JAK1/2 (Janus kinase 1 and 2) etc. [11,12]. Moreover, IL-6 contributes to the malignancy of tumor cells by sustaining the phosphorylation of signal transducer and activator of transcription 3 (STAT3) [13]. Long-term activation of STAT3 is intimately related to the promotion of HCC [14,15]. Target genes of STAT3 (e.g. MCL1 encoding Mcl-1 and CCND1 encoding cyclin D1), are also positive regulators of HCC [16–18]. A previous work indicated that the increased *IL-6* mRNA level correlates to the proliferation and migration in HepG2 cells [19]. Targetting IL-6 leads to the reduction in cell invasion [20]. Above evidence reveal that IL-6/STAT3 signaling pathway and its downstream effectors may play a crucial role in HCC development. However, whether BMSC-CM, a mixture of cytokines containing IL-6, can induce the activation of STAT3 pathway and enhance the invasion ability of HCC cells, still remains unclear.

In the present study, we first performed transwell assays to evaluate the invasion ability of HCC cells that were treated with BMSC-CM, recombinant IL-6, or anti-IL-6 antibody; then we measured the expression of IL-6R, gp130, and STAT3, and assessed the phosphorylation level of STAT3 and the mRNA level of its target genes. These results together demonstrated the function and the regulatory mechanism of BMSC-CM on HCC metastasis; and might shed light on the clinical application of MSC-mediated immunotherapy.

## Materials and methods

### BMSCs separation and culture

Bone marrow aspirates were acquired from healthy donors with signed informed consents. Cells were cultured in DMEM (Invitrogen Life Technologies, Carlsbad, CA, U.S.A.) with 10% FBS (Invitrogen Life Technologies), 100 units/ml penicillin, and 100 μg/ml streptomycin at 37°C in a humidified atmosphere containing 5% CO_2_. Cells were washed with PBS to remove the non-adherent cells after day 3. The medium was changed every 3 days. Cells were passaged when they reached 80% confluence. The morphological features of BMSCs were observed and photographed under an inverted microscope (Nikon Eclipse TE2000-U; Nikon Instruments Inc., Melville, NJ, U.S.A.).

Passage 3–5 BMSCs cultured in 100-mm dishes were washed with PBS thrice and added with a serum-/Phenol Red-free DMEM (Invitrogen Life Technologies). After 2 days, cells reached 90% confluence (approximately 5 × 10^6^ cells per dish). The culture medium (approximately 10 ml per dish) was collected and centrifuged (4000 ***g***, 15 min, 4°C) to remove the debris. These CM were sterilized with 0.22-µm filters (Millipore) and stored at –80°C until use.

### Osteogenic and adipogenic differentiation of MSCs

First, primary MSCs were cultured in high-glucose DMEM (4.5 g/l) for osteogenesis induction or in low-glucose DMEM (1 g/l) for adipogenesis induction, both containing 10% FBS. When grown to 60–80% confluence, the cells were added with pre-prepared adipogenic medium or osteogenic medium. The osteogenesis or adipogenesis differentiation medium was respectively prepared by adding 10 ml StemPro® Osteogenesis Supplement or 10 ml StemPro®Adipogenesis Supplement (Invitrogen Life Technologies) to 90-ml culture medium.

### Alizarin Red S staining

After 3 weeks of growth in osteogenic medium, the cells were washed thrice with PBS, fixed with 4 % formaldehyde for 30 min, and rinsed twice with distilled water. Then, the cells were incubated with 2% Alizarin Red S solution (pH 4.2) for 2 min. Next the cells were washed thrice with distilled water, and images were captured using an inverted microscope (Nikon Eclipse TE2000-U) at 40× magnification.

### Oil Red O staining

After 3 weeks of growth in adipogenic medium, the cells were washed thrice with PBS, fixed with 10 % formaldehyde for 15 min, and rinsed with distilled water. Then the cells were incubated with Oil Red O solution (0.5% in isopropanol, m/v) for 5 min at room temperature. Next, the Oil Red O solution was removed and cells were washed thrice with distilled water, and observed and imaged at 40× magnification under an inverted microscope (Nikon Eclipse TE2000-U).

### Flow cytometry analysis

In addition to the morphological features, BMSCs were identified by the staining of surface antigen CD105, CD44, and CD34 by using flow cytometry analysis. For flow cytometry analyses, approximately 10^6^ BMSCs at passages 3–5 were collected and stained with R-phycoerythrin (PE) mouse antihuman CD105, PE mouse antihuman CD44, FITC mouse antihuman CD34, or an isotype control (BD Biosciences, San Jose, CA, U.S.A.). Labeled cells were acquired on an FACSCalibur flow cytometer with BD CellQuest™ Pro software (BD Biosciences).

### IL-6 assay

The measurement of IL-6 was performed using standardized commercial solid-phase sandwich ELISA (Human IL-6 High Sensitivity ELISA Kit, Abcam, Cambridge, MA, U.S.A.). Briefly, the culture medium of BMSCs was collected and centrifuged (4°C, 1000 ***g***, 10 min) to remove the cell debris. The IL-6 concentration in a volume of 100-μl supernatant was then measured following manufacturer’s instructions.

### Cell culture and treatment

Bel-7404, Bel-7402, and HepG2 cells were obtained from American Type Culture Collection (ATCC, Manassas, VA, U.S.A.) and respectively cultured in RPMI 1640 and DMEM (Invitrogen Life Technologies) with 10% FBS (Invitrogen Life Technologies) at 37°C in a humidified atmosphere with 5% CO_2_.

On reaching 60–80% confluence, both cell lines were treated with BMSC-CM, serum-free DMEM, 2% FBS-containing DMEM, recombinant human IL-6 (Sigma–Aldrich Corp., St. Louis, MO, U.S.A.) or the anti-IL-6 antibody (R&D Systems, Inc., Minneapolis, MN, U.S.A.).

### Western blot analysis

Bel-7404 and HepG2 cells were plated in 60-mm plated and grown to 70% confluence. Cells were treated as indicated, washed with cold PBS, and lysed using standard procedures. Cell lysates were resolved by SDS/PAGE and transferred on to a PVDF membrane. The membrane was blocked with 5% skim milk (m/v) in Tris-buffered saline with Tween-20 (TBST; 50 mM Tris/HCl, pH 7.6, 150 mM NaCl, 0.1% Tween-20) at room temperature for 1 h and incubated with IL-6R, gp130, STAT3, p-STAT3, or tubulin antibodies (Cell Signaling Technology, Beverly, MA, U.S.A.) at 4°C overnight. The membrane was then washed and incubated with horseradish peroxidase (HRP)–conjugated secondary antibodies (Cell Signaling Technology) following exposure to Immobilon™ Western Chemiluminescent HRP Substrate (Millipore, New Orleans, LA, U.S.A.).

### RNA isolation and quantitative RT-PCR

Total RNA was extracted using Invitrogen AmbionTRIzol® LS reagent (Invitrogen Life Technologies) and reverse transcribed using Thermo Scientific Revert Aid First Strand cDNA Synthesis Kit (Thermo Scientific, Waltham, MA, U.S.A.) according to the manufacturer's instructions. qRT-PCR was carried out with FastStart Universal SYBR Green Master (Rox) (Roche FastStart Universal SYBR Green Master (Rox) (Roche Applied Science, Mannheim, Germany) on an ABI Prism 7900 Fast instrument (Applied Biosystems, Foster City, CA, U.S.A.). Reactions were performed in triplicate.

Primers for qRT-PCR reactions were as follows: human IL-6R, forward 5′-TTCTACAGACTACGGTTTGAG-3′ and reverse 5′-GGATGACACAGTGATGCT-3′; human IL-6ST, forward 5′-ACTGTTGATTATTCTACTGTGTAT-3′ and reverse 5′-AATTATGTGGCGGATTGG-3′; human BCL2, forward 5′-GATGACTGAGTACCTGAACC-3′ and reverse 5′-AGTTCCACAAAGGCATCC-3′; human CCND1, forward 5′-CGGAGGAGAACAAACAGA-3′ and reverse 5′-GCGGATTGGAAATGAACTT-3′; human MCL1, forward 5′-CAGGATTGTGACTCTCATT-3′ and reverse 5′-CCTCTACATGGAAGAACTC-3′; human MMP2, forward 5′-ACAAGAACCAGATCACATACAG-3′ and reverse 5′-TCACATCGCTCCAGACTT-3′; human GAPDH, forward 5′-GTCAAGGATTTGGTCGTATT-3′ and reverse 5′-AGTCTTCTGGGTGGCAGTGAT-3′. Then, the mRNA level of *IL-6R, IL-6ST, BCL2, CCND1, MCL1*, and *MMP2* genes was normalized to *GAPDH* mRNA and expressed by 2^−ΔΔ*C*^_T_ (ΔΔ*C*_t_, the relative cyclic value) [21].

### Cell invasion assay

Cell invasion ability was assessed using a 24-well transwell chamber (6.5-mm Transwell® with 8.0 µm pore polyester membrane insert, product #3464, Corning Costar, New York, NY, U.S.A.) covered with Matrigel (BD Biosciences, San Diego, CA, U.S.A.) as described in the manufacturers’ protocol. Cells were treated as indicated and serum-starved for 24 h. Approximately 5 × 10^4^ cells were resuspended in 500 μl FBS-free medium and seeded into the upper wells. The lower wells were added with 500 μl of 20% FBS medium. After 24-h incubation, cells on the upper surface of the filter were removed with a cotton swab. The remaining cells in the lower chambers were washed with PBS, fixed with methanol, and stained with 0.1% Crystal Violet. The invaded cells were photographed and the cell number was counted from at least ten random microscopic central fields.

### Statistical analysis

Data are expressed as mean ± S.E.M. from at least three separate experiments. Statistical analysis of multiple groups was assessed by one-way ANOVA with Turkey’s post-hoc test, and for two groups was calculated by two-tailed Student’s *t* test.

## Results

### Isolation and identification of BMSCs

**Figure 1:**
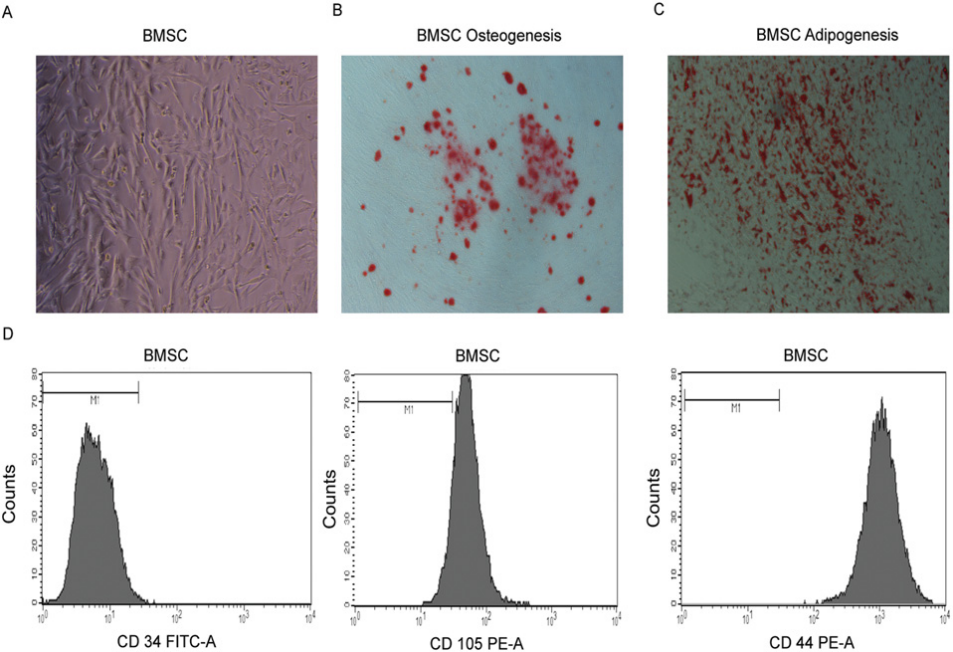
Morphology and identification of human BMSCs. (**A**) Representative cell morphology of BMSCs at day 5. (**B**) Osteogenic differentiationof BMSCs, evident by Alizarin Red S staining. (**C**) Adipogenic differentiation of BMSCs, evident by Oil Red O staining. (**D**) Flow cytometry analysis of BMSCs. BMSCs were negative for CD34, and positive for CD105 and CD44. Magnification: 40× (**A–C**).

The BMSCs were isolated and adhered to the culture plate after seeding for 24 h. The cells become predominantly spindle-shaped after 3 or 4 days (Figure 1A). BMSCs cultured in adipogenic and osteogenic medium differentiated into adipocytes and osteocytes, respectively. Then, Alizarin Red S staining and Oil Red O staining were carried out to detect osteocytes and adipocytes. The captured images showed that a majority of BMSC population can differentiate into osteogenic or adipogenic lineages (Figure 1B,C). The undifferentiated BMSCs were characterized by CD105^+^, CD44^+^, and CD34^−^ (Figure 1 D).

### BMSC-CM promotes HCC cell invasion

**Figure 2:**
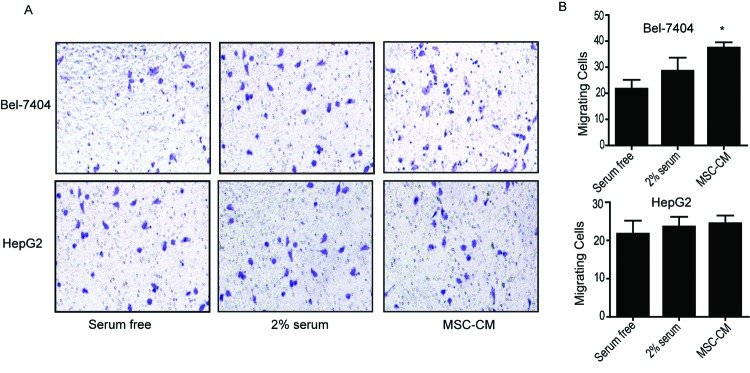
BMSC-CM promotes HCC cell invasion. BMSC-CM was collected and added to Bel-7404 and HepG2 cells. (**A**) Representative images of invading Bel-7404 cells and HepG2 cells that were treated with serum-free medium, 2% FBS containing medium and BMSC-CM. (**B**) The calculated number of invading cells. Data were expressed as mean ± S.E.M. from triplicates; *P<0.05.

**Figure 3:**
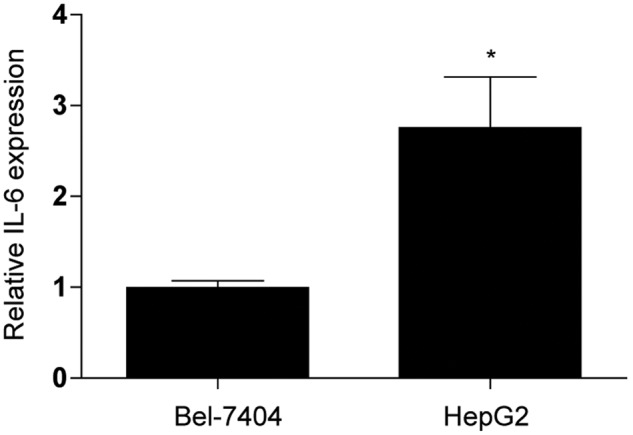
Quantitation of *IL-6* mRNA level in Bel-7404 and HepG2 cells by qRT-PCR. *P<0.05. Data were normalized to *GAPDH* mRNA and expressed by mean ± S.E.M. from triplicates.

First, we detected the IL-6 concentration in BMSC-CM by using ELISA. In accordance with a previous report [10], our study showed that a substantial amount of IL-6 (approximately 589 pg/10^5^ cells, i.e. 2.95 ng/ml) was secreted into the culture medium by BMSC within 48 h (Table 1). To further evaluate the influence of BMSC-CM on HCC cells’ invasion potential, we performed transwell assays on Bel-7404 and HepG2 cells that have been pretreated by BMSC-CM for 24 h (Figure 2). The cells cultured in medium without or with 2% FBS were set as a control or a positive control, respectively. The result showed that BMSC-CM significantly increased the invasion rate of Bel-7404 (*P*<0.05, Figure 2A-B, upper panel) and slightly increased in HepG2 cells (Figure 2A-B, lower panel), compared with the control cells. Notably, as shown in Figure 3 and Table 2, the *IL-6* mRNA level and endogenous IL-6 concentration in HepG2 cells are significantly higher than Bel-7404 cells, suggesting the minor response to exogenous IL-6 might be due to a higher level of endogenous IL-6 in HepG2 cells.

**Table 1:** Quantitation of IL-6 secretion by MSCs

Growth factor	pg/48 h/10^5^ cells (mean ± S.E.M.)
IL-6	589 ± 10.25

Concentration of human BMSC-secreted IL-6. The culture medium was collected 48 h after seeding. The quantitation of IL-6 was performed by ELISA according to the manufacturer’s instructions. The result was expressed as mean ± S.E.M. from at least three independent measurements.

**Table 2:** Quantitation of IL-6 secretion by HepG2 and Bel-7404 cells

Cell line	Growth factor	pg/48 h/10^5^ cells (mean ± S.E.M.)
HepG2	IL-6	212 ± 8.12
Bel-7404	IL-6	35 ± 4.33

Concentration of HCC-secreted IL-6. The culture medium was collected 48 h after seeding. The quantitation of IL-6 was performed by ELISA according to the manufacturer’s instructions. The result was expressed by mean ± S.E.M. from at least three independent measurements.

### BMSC-CM induces the expression of IL-6R and gp130 of HCC cells

**Figure 4:**
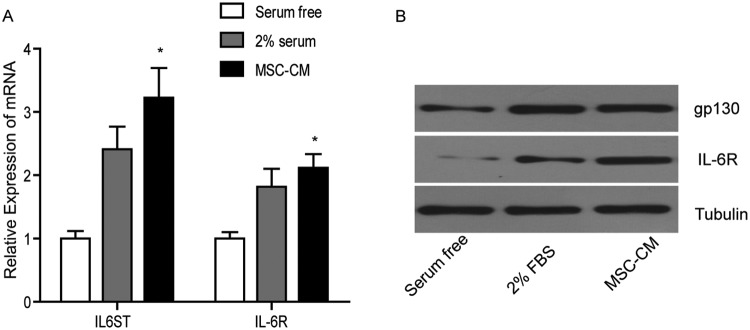
BMSC-CM increases both mRNA and protein levels of gp130 and IL-6R in Bel-7404 cells. (**A**) Quantitative RT-PCR analysis of *IL-6ST* and *IL-6R* mRNA. (**B**) Immunoblotting of IL-6R and gp130; *P<0.05.

To confirm that BMSC-CM secreted IL-6 can induce HCC cell invasion and activate downstream signals in HCC cells, we detected the expression of IL-6R and gp130, the binding partners of IL-6. Quantitative RT-PCR showed that the mRNA levels of IL-6R gene (encoding IL-6R protein) and IL-6ST gene (encoding gp130 protein) in Bel-7404 cells were markedly increased by 1.4- and 0.8-times upon BMSC-CM treatment, compared with that of the control cells (Figure 4A). Similarly, immunoblots showed that the expressions of IL-6R and gp130 protein in Bel-7404 cells were elevated in response to BMSC-CM treatment (Figure 4B). These two proteins were not significantly increased in BMSC-CM-treated HepG2 cells (results not shown). Being insensitive to BMSC-CM treatment of HepG2 cells might be due to the higher endogenous IL-6 level (Table 2). These results proved that the BMSC-CM may stimulate downstream signals of IL-6 by inducing the expression of IL-6R proteins in Bel-7404 cells, which are more sensitive to exogenous IL-6.

### Effects of recombinant IL-6 and anti-IL-6 antibody on HCC cell invasion and downstream signaling pathway activation

**Figure 5:**
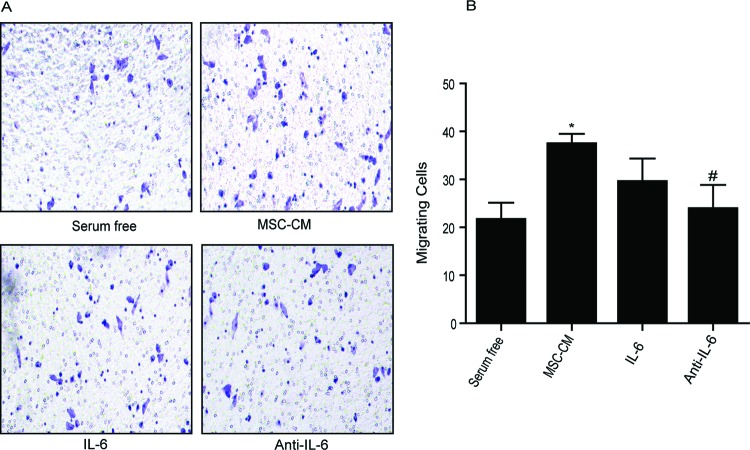
Anti-IL-6 antibody reduces Bel-7404 cell invasion. (**A**) Representative images of invading Bel-7404 cells that treated as indicating. BMSC-CM and recombinant IL-6 promote Bel-7404 cell invasion. Anti-IL-6 antibody significantly reduced cell invasion in BMSC-CM pretreated Bel-7404 cells. (**B**) The calculated number of invading cells. Data were expressed as mean ± S.E.M. from triplicates. ^#^*P*<0.01, compared with BMSC-CM treated cells; *P<0.05, compared with serum-free medium treated cells.

In order to elucidate whether BMSC-CM can induce HCC cell invasion through the IL-6 signaling pathway, we further compared the invasion ability of BMSC-CM-treated, recombinant IL-6 treated and anti-IL-6 antibody treated Bel-7404 cells and HepG2 cells. As shown in Figure 5, both BMSC-CM and recombinant IL-6 (concentration was equal to IL-6 in BMSC-CM) promoted the invasiveness in Bel-7404 cells. When anti-IL-6 antibody was added to cells that were pretreated with BMSC-CM, the invasion rate was significantly decreased by 48%. Similar results were not observed in HepG2 cells (results not shown). Obviously, these results confirmed our assumption that IL-6 secreted by BMSC was responsible for the elevation of cell invasion potential of Bel-7404 but not HepG2 cells. To explore what caused the difference between these two cell lines, we further measured the secretion of IL-6 of another HCC cell line Bel-7402. The secretion of IL-6 from these cells was approximately 26 pg/48 h/10^5^ cells (Supplementary Table S1), even lower than Bel-7404 (Table 2). As expected, the addition of BMSC-CM to Bel-7402 cells significantly promoted cell invasion compared with the untreated control. When the antibody against IL-6 was applied, it diminished cell invasion (Supplementary Figure S1). These data suggested that the BMSC-CM containing high amount of secreted IL-6 may play a role in promoting the Bel-7404 and Bel-7402 cells’ invasion. More interestingly, if we treated the HepG2 cells with a higher amount of recombinant IL-6, the invasion ability was increased in a dose-dependent manner (Supplementary Figure S2). These results showed that the IL-6 in BMSC-CM significantly induced the invasion of HCC cells with a reletively lower endogenous IL-6 such as Bel-7404 and Bel-7402.

**Figure 6:**
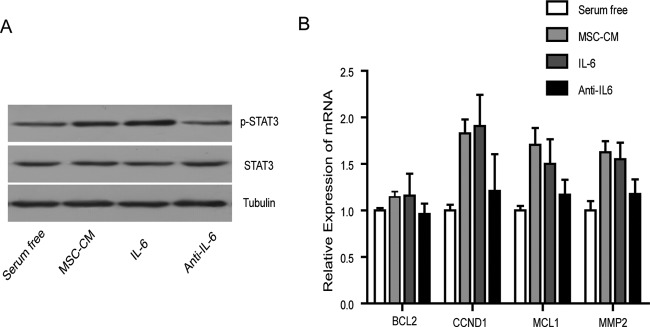
BMSC-CM induces the phosphorylation of STAT3 and the activation of its target genes in Bel-7404 cells. (**A**) Imunoblotting of p-STAT3. BMSC-CM and recombinant IL-6 induced the phosphorylation of STAT3, while anti-IL-6 antibody diminished it. (**B**) Quantitative RT-PCR analysis of BCL2, CCND1, MCL1, and MMP2; *P<0.05.

Next, we analyzed the phosphorylation levels of STAT3 in Bel-7404 cells. The p-STAT3 was induced by BMSC-CM treatment or by recombinant IL-6 treatment. The addition of anti-IL-6 antibody remarkably decreased the p-STAT3 level (Figure 6A). Meanwhile, the phosphorylation of STAT3 was not significantly increased in anti-IL-6 antibody treated cells. Taken together, these results showed that the BMSC-CM may activate IL-6/STAT3 signaling pathway in Bel-7404 cells.

Then, we detected the mRNA levels of four STAT3 target genes, BCL2 (encoding Bcl-2), CCND1 (encoding cyclinD1), MCL1 (encoding Mcl-1), and MMP2 (encoding metalloproteinase-2). The transcription level of these four genes were elevated by BMSC-CM or recombinant IL-6 treatment, and decreased by anti-IL-6 antibody treatment, suggesting the activation of IL-6/STAT3 pathway and its downstream signals in BMSC-CM treated Bel-7404 cells (Figure 6B).

In summary, BMSCs secrete a significant amount of IL-6, which can further induce the activation of IL-6/STAT3 signaling pathways in Bel-7404 cells and promote cell invasion.

## Discussion

MSCs, a heterogeneous population of self-renewable cells with multiple potencies can differentiate into various cell types [22]. They can migrate to inflammatory sites and exert pro-/anti-inflammatory and immunomodulatory functions by interacting with the immune system [23]. They are easily isolated and expanded from several tissues such as bone marrow, adipose tissue, and umbilical cord blood. The clinical applications of MSCs in regenerative medicine have drawn much attention in recent years. [24,25]. Interestingly, researchers noticed that MSCs might be a promising therapeutic approach for cancer treatment [25,26]. They can be home to inflammatory tumorigenic sites and become a key component of the tumor microenvironment [27]. However, controversial evidence accumulated in regard to whether MSCs support or suppress cancer development [1,2].

On one hand, several studies proved that MSCs acted as a useful tool in cancer therapy: the engineered MSCs can precisely release antitumor factors, as they can be home to carcinogenic sites [26,28–31]. For example, Nakamizo et al. [26] have reported that human BMSCs may work as delivery vehicles to release interferon (IFN)-β against glioma cell growth. Qiao et al. [31] have observed that the human MSCs inhibited the proliferation and colony-forming ability of two liver cancer cell lines (H7402 and HepG2). On the other hand, some studies demonstrated that MSCs secrete inflammatory cytokines and promote cancer development [32]. For example, it has been shown that breast cancer cells can stimulate the MSCs within the tumor stroma to produce a chemokine C–C motif chemokine ligand 5 (CCL5). The binding of CCL5 to its specific receptor on breast cancer cells further activates downstream signals to promote the migration and invasion of breast cancer cells [33]. This positive-feedback loop between MSCs and cancer cells implies that the application of MSCs in cancer treatment must be carried out with extreme caution. Another example of the tumor promoting effect of MSC has been reported by Shinagawa et al. [34]. They suggested that MSCs migrate into carcinoma-associated fibroblast and enhance growth and metastasis of colon cancer. These studies illustrated that direct and/or indirect interactions between MSCs and cancer cells stimulate the tumorigenesis and metastasis of multiple types of cancer. However, how MSCs affecting liver cancer, being positive or negative on HCC, is still undetermined.

In the current study, to demonstrate how human BMSCs modulate the tumor microenvironment of HCC, we first employed ELISA to quantitate the concentration of BMSCs-secreted IL-6. The result showed a considerable amount of IL-6 in BMSC-CM (Table 1), suggesting that the BMSC-secreted IL-6 may at least partly contribute to the induction of downstream signals of Bel-7404 and HepG2 cells (Figures 4 and 6). Previous studies have proven that IL-6 can be characterized as a pro-inflammatory cytokine that stimulates immune responses and promotes tumorigenesis and metastasis in a wide range of cancers, e.g. lung cancer, cervical cancer, breast cancer, ovarian cancer, and renal cell carcinoma [35]. For instance, Guthrie et al. [36] have reported that the elevation in circulating IL-6 concentration induced angiogenesis and in turn promoted colorectal cancer progression. Targetting IL-6 signaling pathway may be a promising clinical therapeutic strategy in cancer treatment [37].

In the present study, we found that the ratio of invading HCC cells upon BMSC-CM treatment is comparable with that of recombinant IL-6 treated Bel-7404 cells (Figures 2 and 5). When anti-IL-6 antibody was added to neutralize secreted IL-6 bioactivity, the invasion rate of BMSC-CM pretreated Bel-7404 cells declined accordingly (Figure 5). Surprisingly, the HepG2 cells seemed irresponsive to BMSC-CM treatment (Figure 2). We supposed the reason was the relatively higher mRNA level and concentration of endogenous IL-6 (Figure 3, Supplementary Figures S1 and S2, Table 2, and Supplementary Table S1). If so, these results suggested that when recruited to tumor sites, the MSCs may possibly increase the invasion ability of HCC cells which express lower endogenous IL-6 (such as Bel-7404 and Bel-7402) and therefore, promote its metastasis. A previous study showed a strong correlation between endogenous *IL-6* mRNA level, and the proliferation and migration of HepG2 cells [19]. Inhibiting endogenous IL-6 production, for example by *miR-26a*, which binds to the 3′-UTR of *IL-6* mRNA, can significantly reduce the migration and invasion of HepG2 and some other HCC cells [38]. They also observed that the treatment of IL-6 at a concentration of 25 ng/ml to *miR-26a*-induced HCCLM3 cells rescued the cell-cycle arrest and the invasion blockage. As an HCC cell line with a high IL-6 production, the HepG2 cells showed significant phosphorylation of STAT3 when treated with a surprisingly high concentration of IL-6 at 100 ng/ml [39]. Constantly, the treatment of IL-6 at a concentration of 20 ng/ml significantly promotes the spheroid formation and migration of RNA polymerase II subunit 5 (RPB5)-mediating protein (RMP) knocks down HCCLM3 cells but not the control cells [20]. Since RMP enhances the IL-6 promoter, we assumed that the knockdown of RMP causes a reduction in endogenous IL-6, thus allowing the IL-6 treatment to be effective. Taken together, their results suggested that IL-6 treatment can affect the migration, invasion, or proliferation of HCC cells in which endogenous IL-6 expression is lacking or was inhibited.

In our study, according to the endogenous IL-6 level (Table 2, Supplementary Table S1, Figure 3, Supplementary Figures S1 and S2), the BMSC-CM we used (Table 1, approximately 589 pg/10^5^ cells) can stimulate the invasion and STAT3 signaling pathway of Bel-7404 cells, but not HepG2 cells (Figures 2 and 6), further suggesting that the endogenous cytokine composition markedly affects cell responses to tumor microenvironmental stimuli. For the tumors that secrete a large amount of inflammatory cytokines such as IL-6, the addition of BMSC-CM might potetially benefit the clinical therapy; otherwise the BMSC-CM treatment may induce malignant progression. It would be helpful to evaluate the endogenous IL-6 level before application of BMSC-CM treatment.

To explore the underlying mechanism(s) in BMSC-CM-induced Bel-7404 cell invasiveness, we further detected the key components in IL-6/STAT3 pathway. As expected, the expression levels of IL-6R and gp130 protein, as well as the phosphorylation of STAT3, were elevated by BMSC-CM treatment (Figures 4B and 6A). These results proved that the BMSC-CM treatment can stimulate IL-6/STAT3 signaling pathway, which might contribute to the observed increase in the invasion ability of Bel-7404 cells (Figures 2 and 5). However, the invasion of HepG2 cells is only slightly induced by BMSC-CM due to its higher endogenous IL-6 level (Figure 3 and Table 2) STAT3, a member of the STAT family, is an important transcription factor targetting several genes, such as BCL2, CCND1, MCL1, and MMP2 [40]. The phosphorylation of STAT3 results in the translocation of dimers to nuclear and binding to the promoter region of target genes. STAT3 and its target genes can be activated by cytokines such as IL-6 and IL-22; and they regulate cell growth, differentiation, apoptosis etc. [40,41]. Previous studies demonstrated that STAT3 is intimately associated with cancer development [42,43]. Targetting STAT3 signaling pathway may be a promising clinical therapy for HCC [44]. In accordance with these previous reports, our study suggested that the BMSC-CM treatment activated the STAT3 pathway and transcription of its target genes. However, to verify if this regulation of STAT3 signaling pathway is essential to the BMSC-CM-induced HCC cell invasion ability, further analysis is needed to verify the role and regulation mechanisms of IL-6/STAT3 pathway in BMSC-CM induced tumor progression, such as the shRNA knockdown of STAT3 or the rescue experiment by overexpressing IL-6. The nude mouse xenograft of HCC cells also might strongly support our current results.

In conclusion, human BMSCs contribute, at least partly, to the activation of IL-6/STAT3 signaling pathway and the enhanced invasion of Bel-7404 cells, but not HepG2 cells, suggesting the application of engineered MSC in liver cancer treatment should be evaluated with enormous caution before practice. More experiments should be carried out to measure the interaction between tumor microenvironmental stimuli and the composition of endogenous cytokines and other factors in tumor cells.

## Supporting information

**Table S1.** Concentration of Bel-7402-secreted IL-6. The culture medium was collected 48 h after seeding. The quantification of IL-6 was performed by ELISA assay according to the manufacture’s instruction. The result was expressed by mean ± SEM from at least three independent measurements.

**Figure S1.** Anti-IL-6 antibody reduces Bel-7402 cell invasion. (A) Representative images of invading Bel-7402 cells that treated as indicating. BMSC-CM and recombinant IL-6 promote Bel-7402 cell invasion. Anti-IL-6 antibody significantly reduced cell invasion of BMSC-CM pretreated Bel-7402 cells. (B) The calculated number of invading cells. Data were expressed by mean ± SEM from triplicates. #P<0.01, compared to BMSC-CM treated cells; *P<0.05, **P<0.01, compared to serum free medium treated cells.

**Figure S2.** Recombinant IL-6 promotes HepG2 cell invasion in a dose-dependent manner. (A) Representative images of invading HepG2 cells that treated by different levels of recombinant IL-6. At a concentration of 100 ng, IL-6 significantly promotes the invasion of HepG2 cells. (B) The calculated number of invading cells. Data were expressed by mean ± SEM from triplicates. *P<0.05, compared to serum free medium treated control cells.

## Abbreviations

BMSC: bone marrow mesenchymal stem cell
BMSC-CM: BMSC conditioned medium
CCL5: C–C motif chemokine ligand 5
HCC: hepatocellular carcinoma
HRP: horseradish peroxidase
IL-6: interleukin-6
IL-6R: interleukin-6 receptor
MSC: mesenchymal stem cell
PE: phycoerythrin
RMP: RNA polymerase II subunit 5-mediating protein
STAT3: signal transducer and activator of transcription 3
VEGF: vascular endothelial growth factor
