# Supplementary material for: Secretion of interleukin-6 by bone marrow mesenchymal stem cells promotes metastasis in hepatocellular carcinoma
Source: Biosci Rep. 2017 Jul 21;37(4):BSR20170181. doi: 10.1042/BSR20170181 (PMC5567045; doi:10.1042/BSR20170181)
Supplement: Supplementary file 1 [file bsr-37-bsr20170181-s1.pdf]

Supplementary Material

Secretion of interleukin-6 by bone marrow mesenchymal stem cells promotes metastasis in hepatocellular carcinoma

Table S1 Quantification of IL-6 secretion by Bel-7402

Growth factor	pg/48h/10^5 cells (mean $\pm$ SEM)
IL-6	26 $\pm$ 3.68

Table S1. Concentration of Bel-7402-secreted IL-6. The culture medium was collected 48 h after seeding. The quantification of IL-6 was performed by ELISA assay according to the manufacture's instruction. The result was expressed by mean ± SEM from at least three independent measurements.

Fig.S1

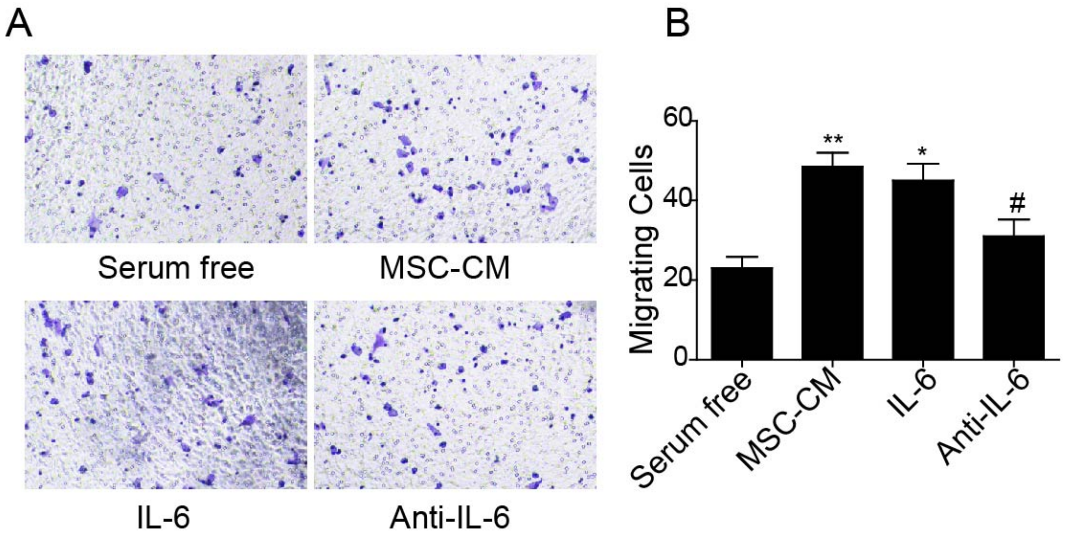

Figure S1. Anti-IL-6 antibody reduces Bel-7402 cell invasion. (A) Representative images of invading Bel-7402 cells that treated as indicating. BMSC-CM and recombinant IL-6 promote Bel-7402 cell invasion. Anti-IL-6 antibody significantly reduced cell invasion of BMSC-CM pretreated Bel-7402 cells. (B) The calculated number of invading cells. Data were expressed by mean ± SEM from triplicates. $^{\#}P<0.01$, compared to BMSC-CM treated cells; $^{*}P<0.05$, $^{**}P<0.01$, compared to serum free medium treated cells.

Fig.S2

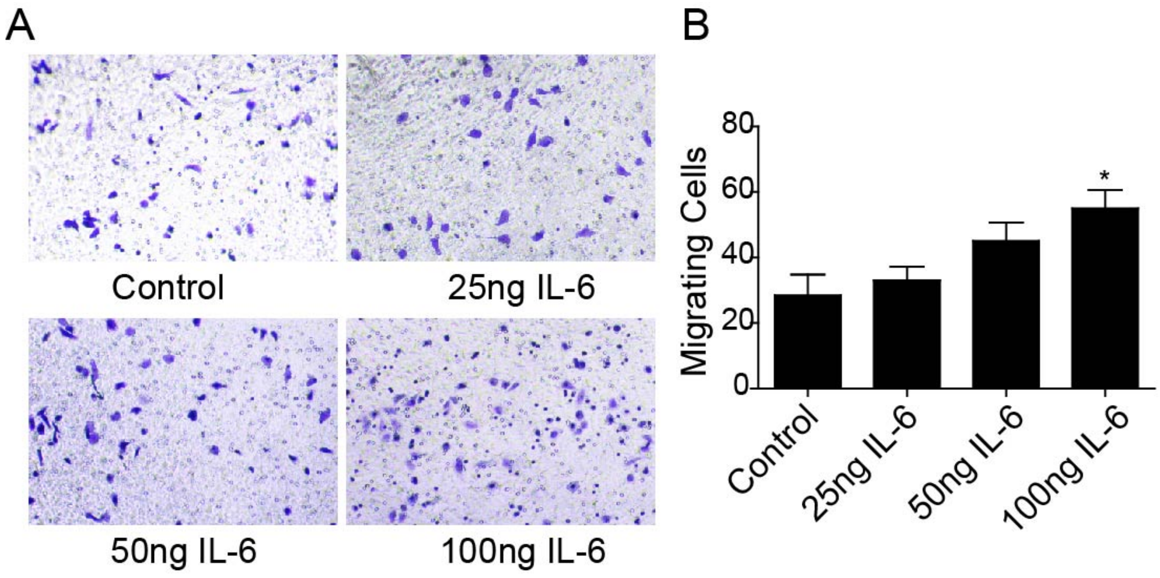

Figure S2. Recombinant IL-6 promotes HepG2 cell invasion in a dose-dependent manner. (A) Representative images of invading HepG2 cells that treated by different levels of recombinant IL-6. At a concentration of 100 ng, IL-6 significantly promotes the invasion of HepG2 cells. (B) The calculated number of invading cells. Data were expressed by mean ± SEM from triplicates. *P<0.05, compared to serum free medium treated control cells.
